# Awareness of Venous Thromboembolism in Patients With Cancer and Their Carers: Protocol for Systematic Review

**DOI:** 10.1111/1759-7714.70093

**Published:** 2025-05-22

**Authors:** Ann‐Rong Yan, Gregory M. Peterson, Mark Naunton, Phillip Newman, Murray R. Turner, Desmond Yip, Nasser Bagheri, Reza Mortazavi

**Affiliations:** ^1^ School of Health Sciences, Faculty of Health University of Canberra Canberra ACT Australia; ^2^ College of Health and Medicine University of Tasmania Hobart TAS Australia; ^3^ Faculty of Health Research Institute of Sport and Exercise, University of Canberra Canberra ACT Australia; ^4^ Faculty of Health University of Canberra Canberra ACT Australia; ^5^ Department of Medical Oncology The Canberra Hospital Garran ACT Australia; ^6^ School of Medicine and Psychology Australian National University Canberra ACT Australia; ^7^ Health Research Institute Faculty of Health, University of Canberra Canberra ACT Australia

**Keywords:** cancer, mixed methods, patient awareness, systematic review, venous thromboembolism

## Abstract

**Background:**

There is an increased risk of venous thromboembolism (VTE) for people living with cancer. However, the awareness of VTE in this population or their carers appears to be low. This lack of awareness may lead to poor outcomes, such as VTE due to low adherence to thromboprophylaxis or delayed hospital presentations with neglected VTE symptoms. This study protocol will guide researchers in conducting a systematic review of existing studies (quantitative, qualitative, or mixed methods) which have assessed VTE awareness among patients living with cancer or their carers. It will also investigate the rates and predictors of VTE awareness in those populations.

**Methods:**

This protocol was designed following the PRISMA‐P 2015 statement and the methodological guidance from the PRISMA 2020 statement. Six databases (APA PsycINFO, CINAHL, Google Scholar, Medline, Scopus, and Web of Science Core Collection) will be searched to retrieve all eligible studies. Covidence software will be utilized to assist in the screening of studies for eligibility. A standard data extraction form will be used, and the Mixed Methods Appraisal Tool (MMAT) will be used to assess the methodological quality of each included study. Both a qualitative descriptive approach and meta‐analysis will be used for data synthesis and integration where possible. The protocol has been registered with PROSPERO (CRD42025628429).

## Graphical abstract

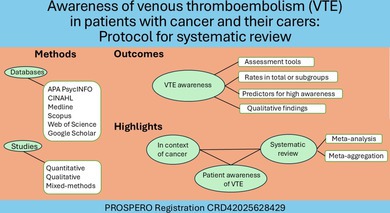

## 1 Introduction

Despite the existence of a strong association between cancer and venous thromboembolism (VTE) [1], awareness and understanding of VTE among patients living with cancer are reportedly low, with one study indicating that only 24% and 15% of patients with cancer were aware of deep vein thrombosis (DVT) and pulmonary embolism (PE), respectively [2]. The pan‐European cancer‐associated thrombosis awareness survey [3], which recruited 1365 participants across six countries, revealed that only 28% of participants with cancer or their carers were aware of VTE [4].

In Australia, improving patient awareness of VTE is a requirement of consumer involvement to meet the Venous Thromboembolism Prevention Clinical Care Standard developed by the Commission on Safety and Quality in Health Care [5], which is integrated into the National Safety and Quality Health Service Standards [6]. Clearly, educating patients about their medical conditions (e.g., the cancer diagnosis and its potential complications, such as VTE) is an important element of any patient‐centered treatment plan to ensure active participation of patients (or their carers) in the delivery of safe and high‐quality care. They should also be assisted to better understand and use the health information they need [7]. Individuals who have been diagnosed with cancer should be aware of the symptoms of VTE and be provided with an action plan in case they notice suspected VTE symptoms. They should also understand the potential benefits and risks of using anticoagulants for the prevention or treatment of VTE.

Lack of awareness or a low understanding of VTE can negatively affect patients' adherence to preventive or therapeutic anticoagulation therapy [8]. On the other hand, enhanced patient awareness through education programs can lead to improved clinical outcomes and quality of life. For instance, the implementaion of a patient education program in France increased the rate of prescribed self‐injection of anticoagulants among patients with cancer, improved the adherence to anticoagulants, and led to improved quality of life among the patients [9]. In another study, conducted by Baddeley et al. an innovative patient education intervention using an informative video at their cancer center, led to a decrease in the amount of time spent from the appearance of VTE symptoms to seeking medical care by an average of six days [10].

On the contrary, the amount and depth of information which should be conveyed to the patient is debatable, as too much information, especially following the cancer diagnosis, may be overwhelming and cause additional stress and emotional burden [11]. Also, there is variation in the pre‐existing knowledge of VTE among individual patients; hence, there is a need for tailored education. In addition to terminology, the knowledge of awareness includes an understanding of VTE risk, VTE signs and symptoms, VTE prevention and treatment, and the associated risk of bleeding imposed by anticoagulants [4]. The source of information, such as patient education programs from doctors and nurses or social media, influences its quality and the effectiveness of gaining accurate and useful knowledge [12, 13, 14].

Therefore, valid assessment instruments are needed in practice to individually assess the knowledge and awareness in detail. The assessment instruments used in this context have typically been surveys [3, 15, 16], although structured questionnaires [13] and semi‐structured interviews [11] have also been used. However, validation results are not commonly available or published for many assessment instruments although most of the surveys and questionnaires were developed and tested by clinical experts [4, 13, 15]. As a prominent example, the survey tested and approved by the European Cancer Patient Coalition (ECPC) has been used in the United Kingdom, France, Germany, Greece, Italy, and Spain [3]. Its domains include knowledge of VTE type, symptoms, diagnosis, prevention, treatment, information source, and VTE risk factors [4]. Another example is the National DVT Awareness Survey in the USA which was designed to evaluate VTE awareness among the general public with one of the four groups studied being oncology patients [15]. Its domains contain awareness of VTE terminology, signs and symptoms, risk factors, patient education, and prevention, but without VTE treatment [15]. Further, a Chinese questionnaire which was developed, validated, and used exclusively in breast cancer surgical patients has comparable domains [14]. Having mentioned all the above, a comprehensive review of the existing instruments for assessing VTE awareness in patients with cancer is necessary to provide a consolidated body of knowledge on patient awareness and needs regarding VTE risk in this context.

Furthermore, some studies have reported potential predictors of VTE awareness, such as sociodemographic characteristics including age, ethnicity, and country/continent, education level [12, 14], and medical history of VTE, surgery, and hospitalization [12, 13, 14]. With this understanding, patient education programs on VTE awareness could be targeted to those with more educational needs [14]. However, the association of some predictors with VTE awareness was reported contradictorily, such as age and education level, which showed a significant relationship with VTE awareness in the study by Wang et al. [14] whereas no such relationship was observed in the study by Souliotis et al. [13] Hence, we also aimed to review reported rates of, and factors associated with VTE awareness in patients with cancer or their carers and provide evidence with a pooled estimate if appropriate.

To the best of our knowledge, there is no published comprehensive review of assessment instruments used for the evaluation of VTE awareness in patients with cancer or their carers. Similarly, no such study was found in the International Prospective Register of Systematic Reviews (PROSPERO) [17]. This study is expected to fill these gaps and provide evidence to inform both clinical practice and future research.

## 2 Aims

The aims of this study are to:

- estimate the rate of awareness of VTE in people with cancer or their carers;
- identify and describe the assessment instruments that have been used to evaluate the awareness of VTE in people with cancer or their carers; and
- identify predictors of awareness of VTE in people with cancer or their carers.

## 3 Methods

This systematic review protocol was structured based on the Preferred Reporting Items for Systematic Reviews and Meta‐analyses for protocols (PRISMA‐P) statement [18]. A completed checklist is available in Table S1. The systematic review will be conducted and reported according to the preferred reporting items for systematic reviews and meta‐analyses (PRISMA) 2020 checklist [19]. The protocol was registered in PROSPERO (registration number CRD42025628429).

### 3.1 Eligibility Criteria

#### 3.1.1 Study Design

The systematic review will include studies examining the awareness of VTE in patients with cancer or their carers. Specific assessment instruments or the ways in which the predictors of the awareness of VTE have been explored will be described, and the results will be used for new information synthesis.

#### 3.1.2 Participants

Studies evaluating whether adults (18 years and older) with cancer or their carers were aware of VTE risk will be included. Studies that were conducted in the general population but had a subgroup of individuals with cancer or their carers and reported the results from the subgroup will also be included. Studies exclusively focusing on health professionals will be excluded.

#### 3.1.3 Outcomes

The main outcomes of this systematic review will be the proportion of the people with cancer or their carers who were aware of VTE, and the assessment tools/instruments used for evaluating the awareness of VTE. An additional outcome will be the predictors of awareness of VTE.

#### 3.1.4 Search Strategy

The APA PsycINFO, CINAHL, Google Scholar, Medline, Scopus, and Web of Science Core Collection databases will be searched for peer‐reviewed articles published in English from database inception to the last day of the search. The search strategy (Table S2) is shown in Supporting Information. Search alerts will be set up for each database, and the searches will be updated 3 months after the first formal search.

#### 3.1.5 Study Selection Process

Two reviewers will independently screen the titles and abstracts of all records returned for potential eligibility (Table 1), with discrepancies resolved by discussion between the team members. Then, two reviewers will independently screen the full text of relevant articles, with disagreements resolved by a third reviewer. The references of the included studies and relevant reviews will be checked for additional studies. The Covidence systematic review software will be used to record all included/excluded studies [20].

**TABLE 1:** Eligibility criteria for this systematic review.

		Inclusion criteria	Exclusion criteria
All studies	Participation (P)	Adults (18 years or older) with cancer or their carers	General population only Healthcare professionals only
Qualitative studies	Phenomena of interest (I)	Studies that mentioned the awareness of VTE, including knowledge and understanding of VTE	Studies of patient experience that only reported attitude, impacts and treatmenta
	Context (Co)	Studies conducted in any setting	None
Quantitative studies	Intervention/exposure (I/E)	Studies that determined the rate of VTE awareness and/or identified factors associated with VTE awareness	Studies focusing solely on patient education programs without assessing awareness
	Comparator (C)	Not applicable	Not applicable
	Outcome (O)	Studies that measure the proportion of people with cancer their carers who were aware of VTE risk Studies that measured predictors associated with VTE awareness	Studies that only measured outcomes unrelated to VTE awareness, such as VTE incidence or risk factors
Other limits		None	Reviews Conference papers/abstracts Opinion papers Letters to the editor Theses Duplicates of studies Full text unavailable Non‐English

#### 3.1.6 Quality Assessment

The mixed methods appraisal tool (MMAT; version 2018) will be used to assess the quality of each included study [21]. The tool considers the sampling strategy and representativeness, appropriateness of quantitative measurements, qualitative and mixed‐method approaches, integration of quantitative and qualitative components in mixed methods, completeness of outcome data, selective reporting, and any other potential bias. Two reviewers will assess the studies independently using Table S3, and discrepancies will be resolved through discussion with a third reviewer.

#### 3.1.7 Data Extraction

Customized data extraction forms provided as Tables S4 and S5 will be developed and piloted by two reviewers with alterations made before data extraction begins. Study characteristics and outcome data will be extracted by one reviewer, and a second reviewer will check data extractions for accuracy. Data to be extracted will include the first author, year of publication, year and period of study, study design, countries and institutions where the data were collected, participant demographic and clinical characteristics (e.g., type of cancer), name and items of each instrument used for awareness assessment, instrument validation status if reported, the awareness rate or level (overall and in any subgroups), the predictors of VTE awareness and their risk ratios, and study limitations. In case of incompletely reported data, study authors will be contacted.

#### 3.1.8 Data Synthesis

A narrative synthesis approach will be used to summarize the characteristics of the range of instruments used for the assessment of VTE awareness.

Meta‐analysis will be performed if there are more than three included studies reporting the same outcome i.e., the proportions of VTE‐aware participants (overall and stratified by specific predictors). If the relevant data are not available, the study will be excluded from meta‐analysis. The Freeman‐Tukey double arcsine transformation will be used to reduce heteroskedasticity for meta‐analysis, and the results are then back transformed to prevalence [22, 23]. The estimation method applied will be a random effect model with prevalence estimate and an effect size measure of risk ratios of the predictors at the 95% confidence interval [24]. If the necessary data are available, subgroup analyses will be undertaken (e.g., for patients with different types of cancer), and meta‐regression analysis will be conducted as well when there are more than 10 study entries. To synthesize the qualitative data, meta‐aggregation will be used to extract all the findings from each included study, develop categories from findings, and then develop synthesized findings from categories [25].

The *I* ^2^ value will be used to evaluate the heterogeneity of the studies (*I* ^2^ < 50% acceptable and *I* ^2^ ≥ 50% moderate to high) [26]. Publication bias will be assessed by using funnel plots with Egger's method if there are 10 or more studies included in the systematic review [27]. A *p* value < 0.05 will be considered statistically significant. RStudio software will be used for statistical analyses.

#### 3.1.9 Strength and Limitations

The protocol was prepared using the PRISMA‐P checklist, and the study methodology was registered with the PROSPERO international database for systematic reviews.

The study findings will improve our understanding of VTE awareness and its predictors in the context of people with cancer or their carers and will guide strategies for further research and enhancing clinical practice.

A notable limitation would be a potentially high heterogeneity among the studies in their methodology (e.g., awareness tools), and cancer types and stages across the studies. The restriction to include only articles published in English may introduce some bias.

## Author Contributions

Conceptualization: A.‐R.Y., G.M.P., M.N., P.N., M.R.T., D.Y., N.B., and R.M. Methodology: A.‐R.Y., G.M.P., M.N., P.N., M.R.T., D.Y., N.B., and R.M. Writing – original draft preparation: A.‐R.Y. Writing – review and editing: A.‐R.Y., G.M.P., M.N., P.N., M.R.T., D.Y., N.B., and R.M. Supervision: G.M.P., M.N., P.N., M.R.T., D.Y., N.B., and R.M. Project administration: A.‐R.Y. All authors have read and agreed to the published version of the manuscript.

## Consent

The authors have nothing to report.

## Conflicts of Interest

The authors declare no conflicts of interest.

## Supporting information


**Data S1.**Supporting Information.

## Data Availability

Data are contained within the article and Supplementary Materials.
