# Supplementary material for: Awareness of Venous Thromboembolism in Patients With Cancer and Their Carers: Protocol for Systematic Review
Source: Thorac Cancer. 2025 May 22;16(10):e70093. doi: 10.1111/1759-7714.70093 (PMC12097841; doi:10.1111/1759-7714.70093)
Supplement: Supplementary file 1 — Data S1.Supporting Information. [file TCA-16-e70093-s001.docx]

Supplementary Table 1 Preferred Reporting Items for Systematic Review and Meta-analysis Protocols (PRISMA-P) 2015 checklist

| Section and topic | Item No. | Checklist item | Reported on page No. |
| --- | --- | --- | --- |
| **Administrative information** | | |  |
| Title: |  |  |  |
| Identification | 1a | Identify the report as a protocol of a systematic review | 1 |
| Update | 1b | If the protocol is for an update of a previous systematic review, identify as such | NA |
| Registration | 2 | If registered, provide the name of the registry (such as PROSPERO) and registration number | 1 |
| Authors: |  |  |  |
| Contact | 3a | Provide name, institutional affiliation, e-mail address of all protocol authors; provide physical mailing address of corresponding author | Title page |
| Contributions | 3b | Describe contributions of protocol authors and identify the guarantor of the review | 5 |
| Amendments | 4 | If the protocol represents an amendment of a previously completed or published protocol, identify as such and list changes; otherwise, state plan for documenting important protocol amendments | NA |
| Support: |  |  |  |
| Sources | 5a | Indicate sources of financial or other support for the review | 5 |
| Sponsor | 5b | Provide name for the review funder and/or sponsor | NA |
| Role of sponsor or funder | 5c | Describe roles of funder(s), sponsor(s), and/or institution(s), if any, in developing the protocol | NA |
| **Introduction** | | |  |
| Rationale | 6 | Describe the rationale for the review in the context of what is already known | 1-3 |
| Objectives | 7 | Provide an explicit statement of the question(s) the review will address with reference to participants, interventions, comparators, and outcomes (PICO) | 3 |
| **Methods** | | |  |
| Eligibility criteria | 8 | Specify the study characteristics (such as PICO, study design, setting, time frame) and report characteristics (such as years considered, language, publication status) to be used as criteria for eligibility for the review | 3, 4, Table 1 |
| Information sources | 9 | Describe all intended information sources (such as electronic databases, contact with study authors, trial registers or other grey literature sources) with planned dates of coverage | 4 |
| Search strategy | 10 | Present draft of search strategy to be used for at least one electronic database, including planned limits, such that it could be repeated | Suppl. Table S2 |
| Study records: |  |  |  |
| Data management | 11a | Describe the mechanism(s) that will be used to manage records and data throughout the review | 4 |
| Selection process | 11b | State the process that will be used for selecting studies (such as two independent reviewers) through each phase of the review (that is, screening, eligibility, and inclusion in meta-analysis) | 4 |
| Data collection process | 11c | Describe planned method of extracting data from reports (such as piloting forms, done independently, in duplicate), any processes for obtaining and confirming data from investigators | 4 |
| Data items | 12 | List and define all variables for which data will be sought (such as PICO items, funding sources), any pre-planned data assumptions and simplifications | 4 |
| Outcomes and prioritization | 13 | List and define all outcomes for which data will be sought, including prioritization of main and additional outcomes, with rationale | 4 |
| Risk of bias in individual studies | 14 | Describe anticipated methods for assessing risk of bias of individual studies, including whether this will be done at the outcome or study level, or both; state how this information will be used in data synthesis | 4 |
| Data synthesis | 15a | Describe criteria under which study data will be quantitatively synthesised | 4, 5 |
|  | 15b | If data are appropriate for quantitative synthesis, describe planned summary measures, methods of handling data and methods of combining data from studies, including any planned exploration of consistency (such as I^2^, Kendall’s τ) | 5 |
|  | 15c | Describe any proposed additional analyses (such as sensitivity or subgroup analyses, meta-regression) | 5 |
|  | 15d | If quantitative synthesis is not appropriate, describe the type of summary planned | 5 |
| Meta-bias(es) | 16 | Specify any planned assessment of meta-bias(es) (such as publication bias across studies, selective reporting within studies) | 5 |
| Confidence in cumulative evidence | 17 | Describe how the strength of the body of evidence will be assessed (such as GRADE) | NA |

Supplementary Table 2 Search strategy

| **Database**: APA PsycINFO (via EBSCOhost) | | |
| --- | --- | --- |
| Search # | Concept | Search terms |
| #1 | Venous thromboembolism | TI (venous-thromb* OR deep-vein-thromb* OR pulmonary-embolism* OR cancer-associated-thromb*) OR AB (venous-thromb* OR deep-vein-thromb* OR pulmonary-embolism* OR cancer-associated-thromb*) |
| #2 | Cancer | TI (cancer* OR malignan* OR neoplasm* OR oncolog*) OR AB (cancer* OR malignan* OR neoplasm* OR oncolog*) |
| #3 | Patient awareness | TI (aware* OR knowledge OR understand* OR education OR patient-information) OR AB (aware* OR knowledge OR understand* OR education OR patient-information) |
| #4 |  | #1 AND #2 AND #3 |
| #5 | Language - English | Limited to |
| **Database**: Web of Science Core Collection | | |
| Search # | Concept | Search terms |
| #1 | Venous thromboembolism | venous-thromb* OR deep-vein-thromb* OR pulmonary-embolism* OR cancer-associated-thromb* |
| #2 | Cancer | cancer* OR malignan* OR neoplasm* OR oncolog* |
| #3 | Patient awareness | aware* OR knowledge OR understand* OR education OR patient-information |
| #4 |  | #1 AND #2 AND #3 |
| #5 | Language - English | Limited to |
| **Database:** Scopus | | |
| Search # | Concept | Search terms |
| #1 | Venous thromboembolism | venous-thromb* OR deep-vein-thromb* OR pulmonary-embolism* OR cancer-associated-thromb* |
| #2 | Cancer | cancer* OR malignan* OR neoplasm* OR oncolog* |
| #3 | Patient awareness | aware* OR knowledge OR understand* OR education OR patient-information |
| #4 |  | #1 AND #2 AND #3 |
| #5 | Language - English | Limited to |
| **Database:** CINAHL Complete (via EBSCOhost) | | |
| Search # | Concept | Search terms |
| #1 | Venous thromboembolism | TI (venous-thromb* OR deep-vein-thromb* OR pulmonary-embolism* OR cancer-associated-thromb*) OR AB (venous-thromb* OR deep-vein-thromb* OR pulmo nary-embolism* OR cancer-associated-thromb*) |
| #2 | Venous thromboembolism [MeSH] | (MH "Venous Thromboembolism") OR (MH "Venous Thrombosis+") OR (MH "Pulmonary Embolism") |
| #3 |  | #1 OR #2 |
| #4 | Cancer | TI (cancer* OR malignan* OR neoplasm* OR oncolog*) OR AB (cancer* OR malignan* OR neoplasm* OR oncolog* ) |
| #5 | Cancer [MeSH] | (MH "Neoplasms+") |
| #6 |  | #4 OR #5 |
| #7 | Patient awareness | TI (aware* OR knowledge OR understand* OR education OR patient-information) OR AB ( aware* OR knowledge OR understand* OR education OR patient-information ) |
| #8 |  | #3 AND #6 AND #7 |
| #9 | Language - English | limited |
| **Database:** Medline (via EBSCOhost) | | |
| Search # | Concept | Search terms |
| #1 | Venous thromboembolism | TI (venous-thromb* OR deep-vein-thromb* OR pulmonary-embolism* OR cancer-associated-thromb*) OR AB ( venous-thromb* OR deep-vein-thromb* OR pulmonary-embolism* OR cancer-associated-thromb* ) |
| #2 | Venous thromboembolism [MeSH] | (MH "Venous Thromboembolism") OR (MH "Venous Thrombosis") OR (MH "Pulmonary Embolism+") |
| #3 |  | #1 OR #2 |
| #4 | Cancer | TI (cancer* OR malignan* OR neoplasm* OR oncolog*) OR AB (cancer* OR malignan* OR neoplasm* OR oncolog*) |
| #5 | Cancer [MeSH] | (MH "Neoplasms+") |
| #6 |  | #4 OR #5 |
| #7 | Patient awareness | TI (aware* OR knowledge OR understand* OR education OR patient-information) OR AB (aware* OR knowledge OR understand* OR education OR patient-information) |
| #8 | Patient awareness [MeSH] | (MH "Awareness”) |
| #9 |  | #7 OR #8 |
| #10 |  | #3 AND #6 AND #9 |
| #11 | Language - English | limited |
| Database: Google Scholar (via Publish or Perish version 8) | | |
| #1 |  | cancer patients awareness venous thromboembolism |
| #2 | The first 500 results | limited |

| Author, year | S1. Are there clear research questions? | S2. Do the collected data allow to address the research questions? | 1. Is the qualitative approach appropriate to answer the research question? | 2. Are the qualitative data collection methods adequate to address the research question? | 3. Are the findings adequately derived from the data? | 4. Is the interpretation of results sufficiently substantiated by data? | 5. Is there coherence between qualitative data sources, collection, analysis and interpretation? | 1. Are the participants representative of the target population? | 2. Are measurements appropriate regarding both the outcome and intervention (or exposure)? | 3. Are there complete outcome data? | 4. Are the confounders accounted for in the design and analysi | 5. During the study period, is the intervention administered (or exposure occurred) as intended? | 1. Is the sampling strategy relevant to address the research question | 2. Is the sample representative of the target population | 3. Are the measurements appropriate? | 4. Is the risk of nonresponse bias low? | 5. Is the statistical analysis appropriate to answer the research question? | 1. Is there an adequate rationale for using a mixed methods design to address the research question | 2. Are the different components of the study effectively integrated to answer the research question? | 3. Are the outputs of the integration of qualitative and quantitative components adequately interpreted | 4. Are divergences and inconsistencies between quantitative and qualitative results adequately addressed? | 5. Do the different components of the study adhere to the quality criteria of each tradition of the methods involved? |
| --- | --- | --- | --- | --- | --- | --- | --- | --- | --- | --- | --- | --- | --- | --- | --- | --- | --- | --- | --- | --- | --- | --- |
|  | Screening questions | | Qualitative | | | | | Quantitative non- randomized | | | | | Quantitative descriptive | | | | | Mixed methods | | | | |
|  |  |  |  |  |  |  |  |  |  |  |  |  |  |  |  |  |  |  |  |  |  |  |

Table 3 Quality of included studies

+ Yes; - No; na Can’t tell

Table 4 Characteristics of the included study

| Author, year | Study design | Population and sample size | Age (years) | Gender | Instrument | | | | |
| --- | --- | --- | --- | --- | --- | --- | --- | --- | --- |
|  |  |  |  |  | Type | Scale | Domain | Number of items* | Validation status, Cronbach’s α coefficient |
|  |  |  |  |  |  |  |  |  |  |

* Items of each instrument if available will be listed in Appendix.

Table 3. Study findings

| Author, year | Rate of awareness about VTE | Source of information | Predictors of higher VTE awareness | Predictors of lower VTE awareness |
| --- | --- | --- | --- | --- |
|  |  |  |  |  |
